# Safety Profile of Gestrinone: A Systematic Review

**DOI:** 10.3390/pharmaceutics17050638

**Published:** 2025-05-11

**Authors:** Vitor Luis Fagundes, Nathália Carolina Barreiro Marques, Amanda Franco de Lima, Alexandre de Fátima Cobre, Fernanda Stumpf Tonin, Raul Edison Luna Lazo, Roberto Pontarolo

**Affiliations:** 1 Programa de Pós-Graduação em Ciências Farmacêuticas, Universidade Federal do Paraná, Curitiba 80210-170, Brazil; vitorluisfagundes@gmail.com (V.L.F.); nathaliamarques1810@gmail.com (N.C.B.M.); amandaklock2308@gmail.com (A.F.d.L.); alexandrecobre@gmail.com (A.d.F.C.); raulunalazo@gmail.com (R.E.L.L.); 2 Health and Technology Research Center (H&TRC), Escola Superior de Tecnologia da Saúde (ESTeSL-IPL), 1990-096 Lisbon, Portugal; fer_stumpf_tonin@hotmail.com; 3 Pharmacy and Pharmaceutical Technology Department, Social and Legal Pharmacy Section, University of Granada, 18012 Granada, Spain; 4 Departamento de Farmácia, Universidade Federal do Paraná, Curitiba 80210-170, Brazil

**Keywords:** adverse effects, anabolic action, gestrinone, hormonal implants, synthetic hormones

## Abstract

**Background:** Gestrinone is a synthetic hormone derived from 19-nortestosterone, exhibiting androgenic, anabolic, anti-progestogenic, and antiestrogenic effects. Gestrinone subcutaneous implants have been used “off label” for aesthetic purposes due to their anabolic action, promoting accelerated metabolism and muscle gain. **Objective:** Our goal is to conduct a systematic review focused exclusively on identifying the safety profile of gestrinone use, without addressing efficacy. **Methods:** This systematic review was performed according to the Joanna Briggs Institute and Cochrane Collaboration recommendations and is reported following the Preferred Reporting Items for Systematic Reviews and Network Meta-Analyses. This article’s searches were carried out in the PubMed, Embase, and Web of Science databases. **Results**: A total of 32 articles were included in this study. The reported adverse events associated with the use of gestrinone were amenorrhea (41.4% of cases), acne, seborrhea (42.7% of reports), decreased libido (26.5%), and hot flushes (24.2%). Other nonspecific symptoms such as hoarseness and cramps were also fairly reported (3.5% and 18.6%, respectively). Other reported effects were associated with breast size reduction (23.7% of patients) and increased transaminases (15.1%). Most studies (40%, n = 24 studies) found significant weight gain (ranging from 0.9 to 8 kg per patient). Abnormalities in bone mineral density were reported in four studies. **Conclusions:** The evidence remains insufficient to fully understand the risks of gestrinone uses associated with its widespread, unregulated use. Thus, further standardized studies and regulatory oversight to ensure patient safety are needed to mitigate potential health risks.

## 1. Introduction

Gestrinone, or R-2323, is a synthetic hormone derived from 19-nortestosterone marketed in Europe, Latin America, and Australia since the 1980s. Initially investigated for its contraceptive potential, its primary approved clinical use is in the treatment of mild to moderate endometriosis (with or without infertility). Additional studies have also explored its application in uterine fibroids [1,2,3]. The antiestrogenic and anti-inflammatory properties of gestrinone contribute to the regression of endometriotic lesions and the reduction of fibroid volume, alleviating associated symptoms such as pelvic pain and abnormal bleeding [4].

Gestrinone primarily binds to progesterone receptors (PRs), exhibiting moderate affinity and anti-progestogenic activity [5]. Although the gestrinone–PR complex is poorly translocated to the nucleus, leading to a predominantly anti-progestogenic effect, some progestomimetic actions have also been observed [5]. By inhibiting endogenous progesterone activity, gestrinone reduces the growth and inflammatory activity of endometrial tissue both in the uterus and in ectopic locations, making it beneficial for treating endometriosis [6,7]. Among the steroid receptors, gestrinone shows the highest binding affinity for androgen receptors (ARs), followed by progesterone receptors (PRs) and, to a lesser extent, estrogen receptors (ERs) [4,5].

In addition to its interaction with PRs, gestrinone binds to androgen receptors (ARs) with relatively high affinity [5], explaining its mild androgenic and anabolic effects. This binding reduces estrogen production since androgens can inhibit aromatase activity, further suppressing endometrial proliferation [4,7,8]. Gestrinone also interferes with the hypothalamic–pituitary–gonadal axis, decreasing the release of gonadotropins and ovarian steroid production, thereby reducing circulating estrogen levels [8,9,10].

Gestrinone binds moderately well to estrogen receptors (ERs) [5]. However, despite occupying ERs, the gestrinone–ER complex does not efficiently activate estrogen-responsive genes [5], contributing to its potent antiestrogenic action. Indeed, gestrinone is recognized for its strong antiestrogenic profile, which is considered its most striking pharmacological feature [4]. This antiestrogenic action is further reinforced by the inhibition of aromatase and modulation of inflammatory pathways, such as through the nuclear factor kappa B (NF-κB) signaling [4].

Moreover, gestrinone displays an affinity for aldosterone and glucocorticoid receptors and antagonist activity against the steroid hormone-binding globulin (SHBG) [4]. These multiple receptor interactions may contribute to additional biological effects observed during clinical use.

The pharmacological profile of gestrinone confers it with interesting metabolic effects, including ovulation suppression, endometrial atrophy, reduction of breast tissue, and a lipolytic effect through inhibiting the adipogenic cascade in adipocytes [8].

When administered orally for contraceptive purposes, gestrinone exhibited anti-gonadotropic and antiestrogenic activity. However, its clinical development in this context was ultimately limited by a narrow therapeutic window and a relatively high incidence of adverse effects such as acne, seborrhea, alterations in lipid profile, and weight gain observed at contraceptive doses [2,11,12]. 

More recently, gestrinone has been explored for aesthetic purposes due to its impact on metabolism and body composition, notably by promoting increased muscle mass and reducing body fat [13]. 

However, the expanded and prolonged use of gestrinone, especially outside approved medical indications, raises concerns regarding potential side effects, given its multi-receptor activity and incomplete safety characterization. Therefore, a systematic review was conducted focused on identifying the safety profile of gestrinone use, without addressing efficacy or restricting the analysis to any specific indication.

## 2. Materials and Methods

This systematic review was performed according to the Joanna Briggs Institute and Cochrane Collaboration [14] recommendations and is reported following the Preferred Reporting Items for Systematic Reviews and Network Meta-Analyses (PRISMA-NMA) [15]. The protocol of this study is available on PROSPERO under the number CRD42024607318. Disagreements were resolved through discussion, with a third author arbitrating in cases of unresolved discrepancies.

### 2.1. Search Strategy and Eligibility Criteria

A comprehensive literature search was conducted in PubMed, Embase, and Web of Science (February 2024) to identify relevant studies to answer the following research question: “What are the potential health effects of using gestrinone?” For the search strategy, synonyms related to “gestrinone” were combined using the Boolean operator OR; no filters or search limitations (e.g., date, language, or study design) were used (supplementary material, Appendix A). Additionally, a search for grey literature was conducted on Google Scholar, excluding patents and citations, to identify studies not indexed in the databases mentioned above. A manual search in the reference lists of the included studies was also performed.

This systematic review included studies meeting the following criteria (PICOS acronym):

- Population: patients using gestrinone;
- Intervention: gestrinone;
- Comparator: placebo or other active drugs;
- Outcome: side effects of gestrinone;
- Study design: clinical trials, randomized or not.

Secondary studies (e.g., reviews), letters to editors, editorials or commentaries, incomplete studies (i.e., lacking information on gestrinone effects), and those written in non-Roman characters were excluded.

### 2.2. Study Selection and Data Extraction

Records retrieved from the databases were exported to the Systematic Review Accelerator (SRA) [16] where duplicates were removed. Study selection phases were conducted using Rayyan web app, free version, Rayyan Systems, Inc. [17]. In the first step, titles and abstracts of the records were independently screened by two authors to remove irrelevant entries. The full texts of potentially eligible studies were then retrieved and independently assessed for eligibility by two of the authors. Finally, studies included in the review had their relevant characteristics extracted in standardized Excel^®^ sheets (Supplementary Material, Table S1). The following information was collected: study baseline characteristics (author, publication date, country, study design, and aim), details of the drug intervention (gestrinone and comparator) (such as number of patients, dosage, route of administration, and treatment duration), and primary outcomes (gestrinone side effects).

### 2.3. Quality Assessment

Two tools were used to assess the methodological quality of included studies: The Risk of Bias 2 (RoB 2) [18] tool and the Risk of Bias in Non-randomized Studies of Interventions tool (ROBINS-I) [19] for randomized and non-randomized studies, respectively. Quality assessment of the included studies was performed independently by 2 reviewers.

The RoB 2 assessed risk of bias across 5 domains: randomization process; deviations from intended interventions; missing outcome data; measurement of the outcome; and selection of the reported result. Its bias risk categories are “low”, “high”, or “some concerns”.

The ROBINS-I included 7 domains that address issues before the start of the interventions, at interventions, and after the start of interventions. The risk of bias in each domain is categorized as low, moderate, serious, or critical. Any disagreements or discrepancies between reviewers were resolved by consensus.

### 2.4. Data Synthesis

A narrative synthesis of the findings from the included studies, structured around population characteristics, geographical region, and gestrinone effects, was developed. Individual results of the studies were summarized as reported by the authors, including types of measures and units. Whenever possible, tables, graphs, and figures were created to improve data interpretation.

## 3. Results

**Figure 1:**
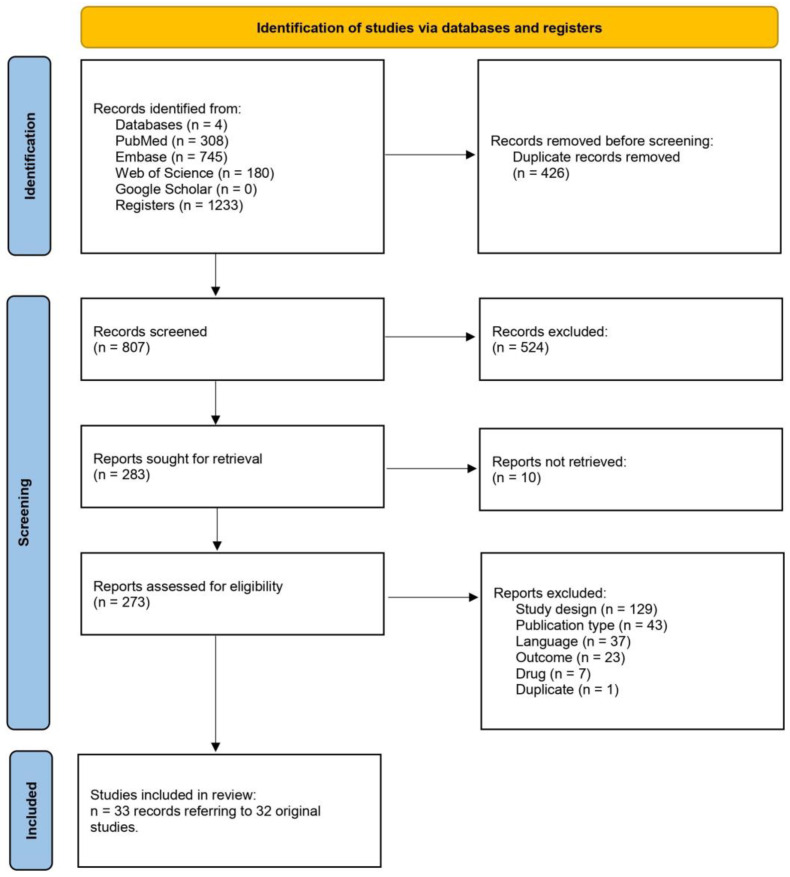
Flowchart of the systematic review.

The systematic search on the three databases (PubMed, Embase, and Web of Science) retrieved 807 records after duplicate removal, of which 524 were considered irrelevant during screening in title and abstract (Figure 1).

From the remaining 273 records assessed in full for eligibility, 240 were excluded. Finally, 33 articles (referring to 32 unique studies, as one is an update [20] of another trial) [21] were included for data extraction and synthesis [3,11,12,20,21,22,23,24,25,26,27,28,29,30,31,32,33,34,35,36,37,38,39,40,41,42,43,44,45,46,47,48,49]. No additional records were identified by manual search. The complete list of excluded studies, along with reasons for exclusion is available in Supplementary Material (Table S2).

**Figure 2:**
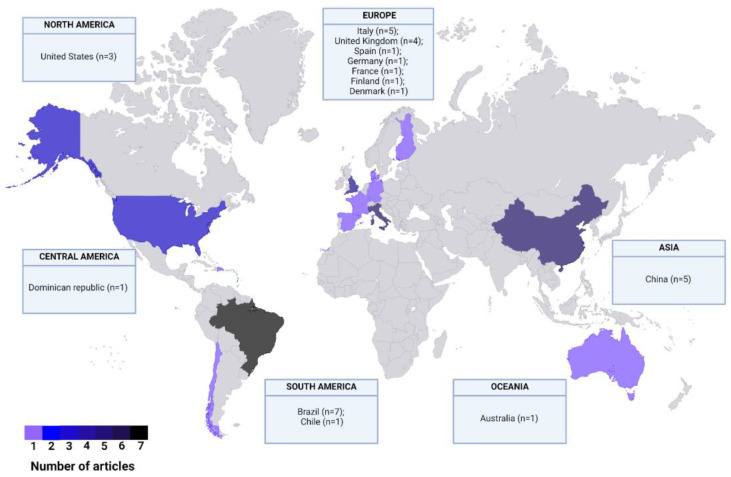
Geographical distribution of the included studies.

Eligible studies were published between 1975 and 2018 and conducted in 14 countries, with the majority from Brazil (*n* = 7; 21.9%), China (*n* = 5; 15.6%), and Italy (*n* = 5; 15.6%) (Figure 2), and were interventional trials involving humans (clinical trials). Twenty of them (62.5%) were randomized, with nine (45.0%) being double-blinded and one (5.0%) single-blinded; however, half of the studies did not have information on blinding (*n* = 10; 50.0%). Although half of the studies (*n* = 16; 50.0%) did not explicitly specify the clinical setting, five (15.6%) were designed as multicentric trials, while eleven (34.4%) were conducted at a single center (see Table 1).

The included articles aimed to assess gestrinone for treatment of endometriosis (*n* = 17; 53.12%), contraception (*n* = 6; 18.75%), as an alternative for uterine leiomyoma (*n* = 3; 9.40%), mastalgia (*n* = 2; 6.25%), fibrocystic disease (*n* = 1; 3.12%), menorrhagia (*n* = 1; 3.12%), pelvic pain associated with endometriosis (*n* = 1; 3.12%), and for preoperative preparation of endometrium (*n* = 1; 3.12%). Thirteen studies reported having funding or conflicts of interest (Table 1).

The thirty-two included articles involved a total of 3745 women, with sample sizes ranging from 11 to 996 participants per study. Participants’ ages ranged from 17 to 45 years. Most studies (*n* = 24; 75.0%) compared the effects of gestrinone with a placebo or other active drugs (levonorgestrel, danazol, buserelin, mifepristone, leuprolide acetate, or triptorelin), while some investigated different doses and administration routes of the interventions. Of the 3745 patients, 61.16% (*n* = 2403) received gestrinone as the intervention (Table 2).

Gestrinone was administered orally (1.25 mg, 2.25 mg, 2.5 mg, or 5.0 mg), subdermally (in the form of implants), and vaginally (Table 3). The most commonly used route of administration was oral, with 1658 patients receiving gestrinone in this manner. Of the 3745 patients, 73 were in the control group and did not receive any drug.

Generally, each of the included studies reported at least one adverse event associated with gestrinone use. Although no consistent pattern of effects was observed, the adverse events most frequently involved the nervous, endocrine, and reproductive systems. The most commonly reported adverse effects were amenorrhea (41.4% of cases), acne and seborrhea (42.7% of reports), decreased libido (26.5%), and hot flushes (24.2%). Other nonspecific symptoms, such as hoarseness and cramps, were reported less frequently (3.5% and 18.6%, respectively) (Table 4).

Other reported effects included breast size reduction (23.7% of patients in eight trials) and increased transaminases (15.1% of patients in four trials), an event indicative of liver damage, as reflected by changes in ALT, AST, and GGT. Two studies reported that around 10% of patients experienced weight loss after using gestrinone [11,49], while three trials observed weight maintenance in about 15% of women [11,24,49]. Conversely, most studies (*n* = 24) found significant weight gain (ranging from 0.9 to 8 kg per patient) associated with gestrinone in approximately 40% of the sample [11,20,21,22,23,25,26,29,30,32,33,34,36,37,38,39,40,41,42,43,48,49].

Only two studies directly compared gestrinone in different pharmaceutical forms and doses, providing safety data separately for each group evaluated [27,49]. Other studies compared gestrinone at different doses but reported only the efficacy results separately by group. At the same time, safety outcomes were aggregated without specifying how many patients in each group developed symptoms [25,28,30,34]. In the study of Coutinho (1988) [49], four different treatment regimens were evaluated (Table 3). It was observed that the group receiving oral gestrinone 2.5 mg had a higher probability, compared to the other groups, of developing the side effects of acne and seborrhea, cramps, and weight gain, reported by 48.15%, 18.52%, and 90.00% of patients, respectively. Interestingly, in the same study, no patient in the group receiving oral gestrinone 2.5 mg developed amenorrhea. Coutinho (1989) [27] found that the three treatment groups showed similar rates of seborrhea: 65.85% (oral gestrinone 2.5 mg), 70.97% (oral gestrinone 5.0 mg), and 64.29% (vaginal gestrinone 5.0 mg). Additionally, only the group receiving oral gestrinone 5.0 mg reported episodes of hot flushes (25.81%). Regarding hirsutism, 12.90% of patients in the oral gestrinone 5.0 mg group and 25.00% of patients in the vaginal gestrinone 5.0 mg group developed this symptom.

Abnormalities in bone mineral density were reported in four studies. In one study [30], a significant bone loss of 7.1% was observed in the group treated with 1.25 mg of gestrinone, while the 2.5 mg gestrinone regimen resulted in a significant bone gain of 7.1%. Another study [34] found no significant changes in bone density in patients treated with gestrinone. A non-significant increase in bone density was reported by one study [43], showing a 0.88% ± 2.12% increase in bone density after six months of treatment. Finally, one study [48] reported an abnormality in bone density in one patient but did not specify whether it was an increase or decrease.

In four studies [29,30,34,42,43], a decrease in cholesterol levels was reported. In three of these studies [29,30,42], there was also a reduction in triglyceride levels. However, it was not specified how many patients these changes occurred. Additionally, a reduction in plasma total thyroxine hormone (T4) was reported in four studies [23,29,34,48]. For instance, one study reported a significant decrease in mean serum thyroxine (T4) in group I from 9.9 ± 2.0 mg/dL at baseline to 7.0 ± 1.1 mg/dL at 6 months (*p* < 0.05) [34]. Another study found that T4 concentrations remained within the same range, although T3 concentrations fell significantly with gestrinone treatment [48]. Additionally, a study noted a lowering of serum T4 without significant changes in T3 [29]. At the same time, another attributed the reduction in plasma total thyroxine concentration to a potential decrease in thyroxine-binding globulin concentration [23].

**Figure 3:**
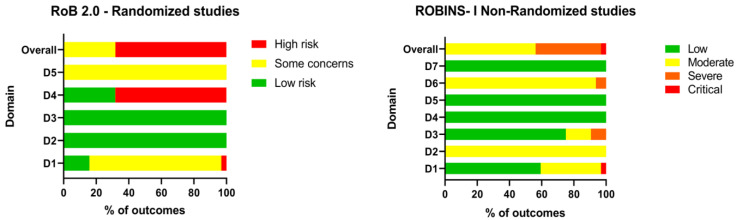
Risk of bias summary (Rob 2.0 and ROBINS-I).

**Figure A1:**
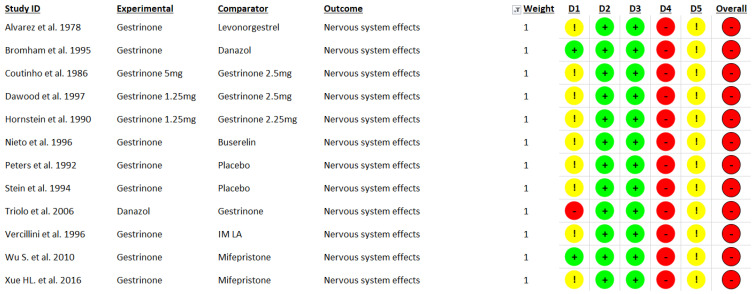
Results of the quality assessment of studies by outcome (RoB-2) (nervous system effects). Legend: risk of bias assessment for each RoB2 domain: randomization process (D1); deviations from the intended interventions (D2); missing outcome data (D3); measurement of the outcome (D4); and selection of the reported result (D5). Risk levels: low risk (green); some concerns (yellow); and high risk (red) [20,21,22,25,30,34,37,38,40,43,44,46,48].

**Figure A2:**
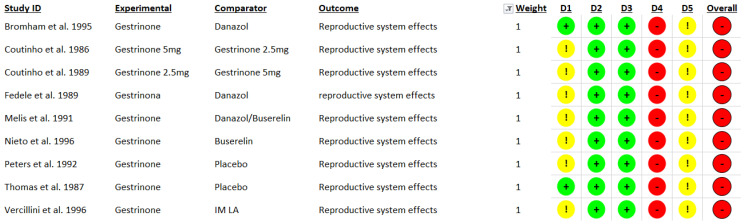
Results of the quality assessment of studies by outcome (RoB-2) (reproductive system effects). Legend: risk of bias assessment for each RoB2 domain: randomization process (D1); deviations from the intended interventions (D2); missing outcome data (D3); measurement of the outcome (D4); and selection of the reported result (D5). Risk levels: low risk (green); some concerns (yellow); and high risk (red) [12,20,21,25,27,32,36,37,38,43].

**Figure A3:**
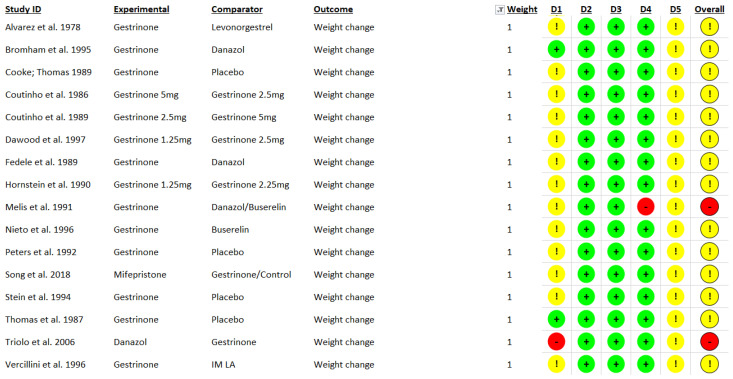
Results of the quality assessment of studies by outcome (RoB-2) (weight change). Legend: risk of bias assessment for each RoB2 domain: randomization process (D1); deviations from the intended interventions (D2); missing outcome data (D3); measurement of the outcome (D4); and selection of the reported result (D5). Risk levels: low risk (green); some concerns (yellow); and high risk (red) [12,20,21,23,25,27,30,32,34,36,37,39,40,43,48].

**Figure A4:**
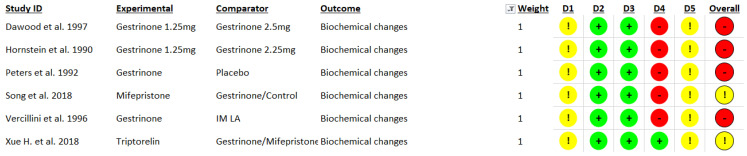
Results of the quality assessment of studies by outcome (RoB-2) (biochemical changes). Legend: risk of bias assessment for each RoB2 domain: randomization process (D1); deviations from the intended interventions (D2); missing outcome data (D3); measurement of the outcome (D4); and selection of the reported result (D5). Risk levels: low risk (green); some concerns (yellow); and high risk (red) [30,34,39,45,48].

**Figure A5:**
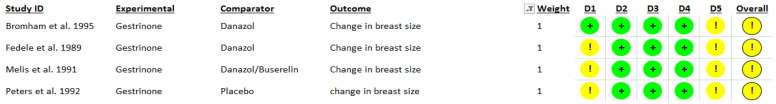
Results of the quality assessment of studies by outcome (RoB-2) (change in breast size). Legend: risk of bias assessment for each RoB2 domain: randomization process (D1); deviations from the intended interventions (D2); missing outcome data (D3); measurement of the outcome (D4); and selection of the reported result (D5). Risk levels: low risk (green); some concerns (yellow); and high risk (red) [20,21,32,36,48].

**Figure A6:**
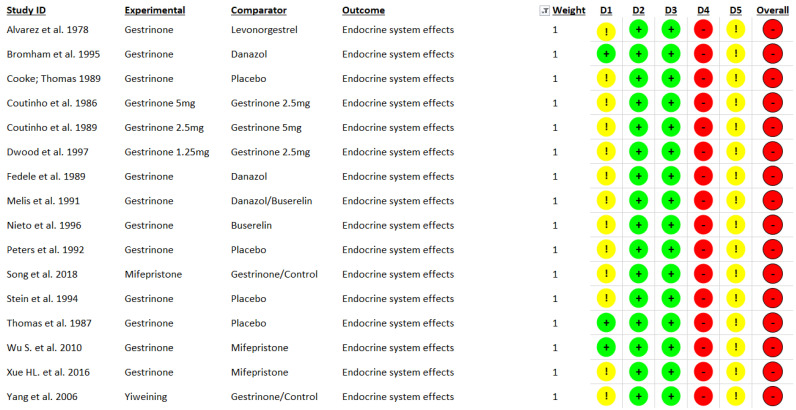
Results of the quality assessment of studies by outcome (RoB-2) (endocrine system effects). Legend: risk of bias assessment for each RoB2 domain: randomization process (D1); deviations from the intended interventions (D2); missing outcome data (D3); measurement of the outcome (D4); and selection of the reported result (D5). Risk levels: low risk (green); some concerns (yellow); and high risk (red) [12,20,21,22,23,25,27,30,32,36,37,38,39,44,46,47,48].

The risk of bias for the included articles was assessed using the RoB 2.0 and ROBINS-I tools and summarized (Supplementary Material, Appendix B and Appendix C). The quality of the studies was assessed by the outcome, with the five outcomes assessed being the following: side effects on the nervous (Appendix B, Figure A1), endocrine, and reproductive systems (Appendix B, Figure A2 and Figure A6), changes in body weight (Appendix B, Figure A3), and changes in breast size and biochemical changes (Appendix B, Figure A4 and Figure A5). Not all studies had all outcomes. The overall bias of the 21 randomized studies assessed by RoB 2.0, by outcome, was considered to have “some concerns” (31.75%) and “high risk” (68.25%). When assessing the risk of bias using the ROBINS-I tool for the 12 non-randomized studies, the overall risk for outcomes was classified as moderate (56.25%), severe (40.63%), and critical (3.13%) (Figure 3) (refer to Appendix B and C for the detailed results by outcomes and risk of bias domains).

## 4. Discussion

This systematic review gathered evidence from 32 clinical trials on the safety profile of the synthetic hormone gestrinone (originally developed for managing specific medical conditions, e.g., endometriosis, uterine fibroids, and as a contraceptive). Gestrinone’s current off-label use has prompted statements from healthcare professionals and public health authorities [10]. In fact, the illicit use of anabolic–androgenic steroids (AAS), such as gestrinone, for aesthetic purposes and by athletes, such as competitive bodybuilders, has increased significantly worldwide over the last three decades. This use is often justified by perceived benefits, including increased muscle mass, weight loss, improved physical disposition, and enhanced skin appearance [50,51,52]. However, as confirmed in this study, scientific evidence supporting these effects is scarce, which may have influenced regulatory decisions regarding the prohibition of gestrinone for non-therapeutic purposes by international authorities, such as the World Anti-Doping Agency (WADA), and in certain countries, including Australia, Argentina, Brazil, the United Kingdom, and the United States [53,54].

Yet, the number of gestrinone consumers has not decreased, likely due to the convenience of online purchasing, unregulated pharmacies that often provide uncertain quality products at lower prices, and insufficient regulation monitoring the consumption of this substance by the population. Additionally, the lack of well-designed clinical trials assessing appropriate drug dosage and regimen, coupled with inconsistent reporting of safety outcomes, hinders a thorough understanding of the true effects of this hormone [55].

Although it was not possible to verify the correlation between the intensity of gestrinone’s effects administered through different forms (subdermal implants, oral pills, and intravaginal doses) and the doses—due to the limited number of studies with sufficient and detailed data—the available evidence suggests that some adverse effects were more prevalent in groups receiving specific doses. However, the data are still too limited and heterogeneous to allow definitive conclusions regarding the safety profile of different doses or routes of administration. Therefore, more standardized and recent studies directly comparing different doses and administration routes of gestrinone are needed to determine better which regimens are associated with a lower incidence of adverse effects.

The scientific literature highlights the importance of rigorous quality control for these products at the manufacturing site to mitigate health risks. Substances available in the form of transdermal drug delivery systems have frequently been associated with quality problems, primarily concerning drug release and absorption, leading to product recalls and discontinuation [56,57]. Moreover, gestrinone subdermal implants require an additional medical procedure for insertion, which is often unregulated and performed clandestinely, leading to further risks, including complications and infections.

Most of the studies gathered in our review were published in Brazil and several European countries, where the prevalence of AAS use is notably high [51]. Although all patients involved in the studies were female—likely due to the therapeutic indications and goals of the trials—men still constitute the majority of AAS consumers worldwide [52]. However, despite an extensive systematic search, no clinical evidence was found regarding the safety of gestrinone in men. This absence may be explained by the fact that gestrinone was initially developed for the treatment of conditions affecting the female reproductive system. Although including studies conducted in male populations could have enriched this review, such data are not yet available in the literature. A meta-analysis of 187 studies providing data on 271 lifetime prevalence rates indicated an overall AAS use rate of 3.3%, which is four times higher in males than in females (6.4% vs. 1.6%, respectively) [51,58]. Moreover, about 30% of AAS users are at risk of developing dependence, leading to significant challenges related to dependency treatment and withdrawal symptoms (e.g., depression, insomnia, suicidal thoughts, and fatigue), which may persist for months [59,60]. This highlights the need to broaden the assessment of the impact of gestrinone and other AAS across different populations (e.g., men, children/adolescents, and individuals with comorbidities) and over longer follow-up periods (e.g., real-world studies) to provide further evidence on the matter.

We found that gestrinone is associated with significant adverse events, including amenorrhea, acne and seborrhea, weight gain, abnormalities in bone mineral density, and changes in biochemical parameters. According to the European Medicines Agency’s definition of side effects frequency, these events (occurring in around 40% of cases) can be classified as common—occurring in 1 to 10 patients per 100. The remaining 60% are very common effects, occurring in more than 10 patients per 100 [61]. Bone density abnormalities are a significant concern, usually related to deficiencies in testosterone and estrogen [62]. Gestrinone interacts with estrogen receptors, reducing the binding of sex hormone-binding globulin (SHBG) to testosterone in the plasma [52]. This increases the proportion of biologically active free testosterone, contributing to its androgenic activity. However, reductions in bone density are often difficult to detect clinically until a fracture occurs due to decreased bone strength. Hip fractures, one of the most common events in women, are associated with significant morbidity. Approximately 50% of patients experience a reduced ability to live independently, with mortality rates reaching 20% [63].

Due to the lack of safety and efficacy studies, some organizations have banned the use and commercialization of gestrinone. The World Anti-Doping Agency (WADA) has included gestrinone on its list of prohibited substances, classifying it as an anabolic agent [64]. In Brazil, the Brazilian Health Regulatory Agency has issued a resolution prohibiting the advertisement of gestrinone and products containing it to the public [65]. The continued use of this hormone in the manner it has been applied poses a public health risk.

The adverse events reported in clinical trials are consistent with the pharmacological mechanisms of gestrinone. As a synthetic steroid, gestrinone exhibits mixed agonist and antagonist activity at progesterone and androgen receptors, in addition to antiestrogenic properties. By inhibiting gonadotropin secretion, suppressing endogenous estrogen and progesterone production, and increasing free testosterone levels through SHBG displacement, gestrinone leads to significant hormonal imbalance. This mechanism explains the frequent occurrence of amenorrhea, acne, seborrhea, reduced libido, hot flushes, and breast atrophy observed in patients. It is important to note that although amenorrhea is commonly reported as an adverse event, its occurrence is often expected—and sometimes even therapeutically desirable—given that gestrinone acts as a progestogen and suppresses ovulatory cycles. Moreover, the modulation of androgen receptors and reduction of estrogenic activity contribute to bone density abnormalities, changes in lipid profiles, and potential hepatic enzyme elevations. While detrimental in therapeutic contexts, these pharmacodynamic characteristics have been exploited for aesthetic purposes under the misconception that gestrinone could promote muscle mass gain and body fat reduction. However, the risks associated with hormonal dysregulation, including long-term metabolic and cardiovascular complications, outweigh any perceived short-term benefits. Therefore, the off-label use of gestrinone as a “beauty chip” lacks scientific support and represents a significant health hazard.

Several adverse effects observed with gestrinone use, such as acne, seborrhea, hirsutism, and weight gain, are consistent with known side effects of progestins [66]. Although the data available for gestrinone derives primarily from observational studies and clinical trials with limited methodological rigor, these effects are commonly attributed to the action of synthetic progestogens in general. Notably, irregular uterine bleeding is a frequent event among users of continuous progestin therapy. At the same time, weight gain, depressive symptoms, and alterations in lipid profiles are also described, especially with long-term use [67]. Additionally, bone density abnormalities are less commonly reported with traditional progestins, which parallels the findings of bone mineral density changes reported in some gestrinone studies. Therefore, the adverse events identified in this review align with the known profile of synthetic progestins and highlight the compounded risks arising from the androgenic and antiestrogenic activities specific to gestrinone.

The off-label use of anabolic steroids has sparked debate within medical and pharmaceutical communities. On one side, some doctors advocate for their use, while others treat patients affected by the misuse of hormones like gestrinone. The Federal Council of Medicine in Brazil addressed this issue with resolution no. 2333/23, highlighting the growing concern over the aesthetic use of these substances. The resolution underscores the lack of significant benefits and the potential adverse effects that jeopardize health [68].

In Brazil, compounding pharmacies have been producing the so-called “beauty chip” without proper authorization from regulatory agencies. As these products are not regulated, it is uncertain whether they undergo effective quality control, further increasing the risks. A recent case involved a 20-year-old woman who was hospitalized with cerebral edema about 24 h after the insertion of implants containing oxytocin, testosterone, gestrinone, and cyproterone [69]. This case illustrates the potential risks of indiscriminate use and highlights that the dangers associated with hormonal implants, such as “beauty chips”, are not disclosed with the same emphasis as their purported benefits. Consequently, it raises concerns about how well-informed users are about the potential risks and the still not fully understood adverse effects.

This study has several limitations. First, studies written in non-Roman characters were excluded, which may introduce a language bias in the findings. However, the included trials are well-distributed geographically, following the expected pattern of scientific publications. Additionally, despite the limited number of studies addressing the safety and efficacy of gestrinone, most of these studies are outdated, and many lack numerical data on the frequency of specific side effects, only indicating whether the effects occurred or not. Another limitation is that, although most of the studies included in the review were conducted in controlled clinical settings, not all specified the origin of the gestrinone. Out of the 32 studies analyzed, only 17 reported the source of the compound, and all used products of industrial origin, likely with pharmaceutical quality control. However, the lack of this information in the other studies prevents the exclusion of variations in the origin and quality of the products used, which could represent a relevant source of heterogeneity when considering the reported adverse events. Furthermore, all the data reported in this review pertain to the safety of gestrinone in women, which limits our understanding of its overall safety.

## 5. Conclusions

The synthesized data from available clinical trials provide valuable insights into the safety profile of gestrinone; however, the evidence remains insufficient to fully understand the risks associated with its widespread, unregulated use. This review highlights concerns about the safety profile of gestrinone, particularly at usual doses, and raises further alarms regarding higher doses, such as those used in implants. Notably, no studies specifically evaluating its off-label use were included, despite its common utilization for purposes like weight loss-an application contradicted by findings indicating potential weight gain.

The lack of high-quality, up-to-date evidence on gestrinone’s indications, particularly as an anabolic agent or in non-approved uses, underscores the need for further standardized research. Such studies should properly assess clinical outcomes and guide safer practices. Additionally, enhanced regulatory oversight is crucial to mitigate potential health risks associated with the off-label and unsupervised use of this hormone.

## Figures and Tables

**Table 1:** Overall characteristics of the 32 studies included in this review.

Authors (year)	Aim	Funding	Conflict of Interest	Setting	Study Design
(Alvarez et al., 1978) [22]	Contraception	Yes	NR	Multicenter	Randomized, without blinding information
(Bromham et al., 1995) [20,21]	Endometriosis treatment	NR	NR	Multicenter	Randomized, double-blind
(Cooke & Thomas, 1989) [23]	Endometriosis treatment	NR	NR	NR	Randomized, double-blind
(Coutinho, 1982) [24]	Endometriosis treatment	Yes	NR	Unicenter	Single arm trial
(Coutinho, 1990) [11]	Uterine leyomas treatment	NR	NR	NR	Single arm trial
(Coutinho & Azadian-Boulanger, 1988) [49]	Endometriosis treatment	NR	NR	Unicenter	Single arm trial
(Coutinho & Azadian-Boulanger, 1984) [26]	Fibrocystic disease treatment	Yes	NR	NR	Single arm trial
(Coutinho et al., 1986) [25]	Uterine leyomas treatment	NR	NR	NR	Randomized, without blinding information
(Coutinho EM et al., 1975) [28]	Contraception	Yes	NR	NR	Single arm trial
(Coutinho & Goncalves, 1989) [27]	Uterine leyomas treatment	Yes	NR	NR	Randomized, without blinding information
(David et al., 1979) [29]	Contraception	NR	NR	NR	Single arm trial
(Dawood et al., 1997) [30]	Endometriosis treatment	Yes	NR	Unicenter	Randomized, double-blind
(Diaz et al., 1977) [31]	Contraception	Yes	NR	NR	Single arm trial
(Fedele et al., 1989) [32]	Endometriosis treatment	NR	NR	NR	Randomized, without blinding information
(Forbes & Thomas, 1993) [33]	Endometriosis treatment	NR	NR	NR	Open-label
(Hornstein et al., 1990) [34]	Endometriosis treatment	Yes	NR	Unicenter	Randomized, double-blind
(Kauppila et al., 1985) [35]	Endometriosis treatment	Yes	NR	NR	Single arm trial
(Melis et al., 1991) [36]	Endometriosis treatment	NR	NR	NR	Randomized, without blinding information
(Nieto et al., 1997) [37]	Endometriosis treatment	NR	NR	Unicenter	Randomized, without blinding information
(Peters, 1992) [48]	Mastalgia	NR	NR	Multicenter	Randomized, double-blind
(Azadian-Boulanger et al., 1976) [3]	Contraception	NR	NR	Unicenter	Single arm trial
(Song et al., 2018) [39]	Endometriosis treatment	NR	NR	Unicenter	Randomized, without blinding information
(Peters et al., 1994) [38]	Mastalgia	NR	NR	Unicenter	Randomized, double-blind
(Thomas & Cooke, 1987) [12]	Endometriosis treatment	Yes	NR	NR	Randomized, double-blind
(Triolo et al., 2006) [40]	Preoperative endometrial preparation	NR	NR	NR	Randomized, open-label
(Turnbull & Rebs, 1990) [41]	Menorrhagia treatment	NR	NR	Unicenter	Randomized, single-blind
(Venturini et al., 1989) [42]	Endometriosis treatment	NR	NR	NR	Single arm trial
(Vercellini et al., 1996) [43]	Pelvic pain associated with endometriosis	Yes	NR	Multicenter	Randomized, double-blind
(Wu et al., 2010) [44]	Contraception	Yes	NR	Multicenter	Randomized, double-blind
(Xue et al., 2016) [46]	Endometriosis treatment	No	No	Unicenter	Randomized, single-blind
(Xue et al., 2018) [45]	Endometriosis treatment	No	No	Unicenter	Randomized, without blinding information
(Yang et al., 2006) [47]	Endometriosis treatment	Yes	NR	NR	Randomized, without blinding information

NR: not reported.

**Table 2:** Intervention characteristics.

Interventions	Pharmacological Classification	(%) of Patients
Gestrinone	Progestin	2403 (64.16%)
Levonorgestrel	Progestin	52 (1.39%)
Danazol	Synthetic androgen	244 (6.51%)
Buserelin	GnRH agonists	28 (0.75%)
Placebo	Inactive substance	164 (4.38%)
Mifepristone	Antiprogestin	683 (18.24%)
Control	-	73 (1.95%)
Triptorelin	GnRH agonists	50 (1.33%)
Leuprolide acetate	GnRH agonists	28 (0.75%)
Yiweining	Herbal medicine	20 (0.53%)
Total number of patients	-	3745 (100%)

**Table 3:** Treatment characteristics.

Authors (Year)	Intervention	N	Route of Administration	Dose	Dosage	Duration of Treatment
(Alvarez et al., 1978) [22]	Gestrinone	48	Subdermal	30.1 ± 1.2 mg	-	504.2 months
	Levonorgestrel	52	Subdermal	29.7 ± 0.9 mg/33.9 ± 0.7 mg	-	512.5 months
(Bromham et al., 1995) [20,21]	Gestrinone	132	Oral	2.5 mg	Twice weekly	6 months
	Danazol	137	Oral	200 mg	Twice daily	6 months
(Cooke & Thomas, 1989) [23]	Gestrinone	18	Oral	2.5 mg	Twice weekly	6 months
	Placebo	17	Oral	-	Twice weekly	6 months
(Coutinho, 1982) [24]	Gestrinone	20	Oral	5 mg	Twice weekly	A period of 6 to 8 months
(Coutinho, 1990) [11]	Gestrinone	24	Oral	5 mg	Three times weekly	A period of 6 to 1 year
(Coutinho & Azadian-Boulanger, 1988) [49]	Gestrinone	17	Vaginal	2.5 mg	Three times weekly	6 months
	Gestrinone	30	Vaginal	2.5 mg	Twice weekly	7 months
	Gestrinone	30	Vaginal	5 mg	Twice weekly	8 months
	Gestrinone	27	Oral	2.5 mg	Twice weekly	9 months
(Coutinho & Azadian-Boulanger, 1984) [26]	Gestrinone	28	Oral	5 mg	Twice weekly	3–9 months
(Coutinho et al., 1986) [25]	Gestrinone	34	Oral	5 mg	Twice weekly	4 months
	Gestrinone	36	Oral	2.5 mg	Three times weekly	4 months
	Gestrinone	27	Vaginal	2.5 mg	Three times weekly	4 months
(Coutinho EM et al., 1975) [28]	Gestrinone	98	Subdermal	30–40 mg	2 implants	9 months
	Gestrinone	180	Subdermal	30–40 mg	3 implants	9 months
	Gestrinone	181	Subdermal	30–40 mg	4 implants	9 months
	Gestrinone	68	Subdermal	30–40 mg	5 implants	9 months
(Coutinho & Goncalves, 1989) [27]	Gestrinone	41	Oral	2.5 mg	Three times weekly	3 months
	Gestrinone	31	Oral	5.0 mg	Three times weekly	3 months
(David et al., 1979) [29]	Gestrinone	28	Vaginal	5.0 mg	Three times weekly	3 months
	Gestrinone	28	Oral	5.0 mg	Once weekly	3 months
(Dawood et al., 1997) [30]	Gestrinone	5	Oral	1.25 mg	Twice weekly	6 months
	Gestrinone	6	Oral	2.5 mg	Twice weekly	6 months
(Diaz et al., 1977) [31]	Gestrinone	38	Subdermal	30.91 ± 1.19 mg	-	-
(Fedele et al., 1989) [32]	Gestrinone	20	Oral	2.5 mg	Twice weekly	6 months
	Danazol	19	Oral	600 mg	Once daily	6 months
(Forbes & Thomas, 1993) [33]	Gestrinone	12	Oral	2.5 mg	Twice weekly	24 weeks
	Danazol	11	Oral	400 and 800 mg	Once daily	24 weeks
(Hornstein et al., 1990) [34]	Gestrinone	6	Oral	1.25 mg	Twice weekly	6 months
	Gestrinone	6	Oral	2.25 mg	Twice weekly	6 months
(Kauppila et al., 1985) [35]	Gestrinone	11	Oral	2.5 mg	Twice weekly	-
(Melis et al., 1991) [36]	Gestrinone	10	Oral	2.5 mg	Twice weekly	6 months
	Danazol	10	Oral	200 mg	Three times daily	6 months
	Buserelin	10	Subcutaneously	300 ug	Three times daily	6 months
(Nieto et al., 1997) [37]	Gestrinone	25	Oral	2.5 mg	Twice weekly	6 months
	Buserelin	18	Intranasal	300 ugrs	Three times daily	6 months
(Peters, 1992) [48]	Gestrinone	73	Oral	2.5 mg	Twice weekly	3 months
	Placebo	72	Oral	-	-	3 months
(Azadian-Boulanger et al., 1976) [3]	Gestrinone	181	Oral	2.5 mg	Once weekly	2 to 44 months
(Song et al., 2018) [39]	Mifepristone	60	Oral	10 mg	Once daily	6 months
	Gestrinone	60	Oral	2.5 mg	Twice weekly	6 months
	Control	60		-	-	-
(Peters et al., 1994) [38]	Gestrinone	38	Oral	2.5 mg	Twice weekly	3 months
	Placebo	40	Oral	-	Twice weekly	3 months
(Thomas & Cooke, 1987) [12]	Gestrinone	18	Oral	2.5 mg	Twice weekly	24 weeks
	Placebo	17	Oral	-	Twice weekly	24 weeks
(Triolo et al., 2006) [40]	Danazol	67	Oral	200 mg	Three times daily	4 or 5 weeks
	Gestrinone	69	Oral	2.5 mg	Twice weekly	5 or 5 weeks
(Turnbull & Rebs, 1990) [41]	Gestrinone	19	Oral	2.5 mg	Twice weekly	12 weeks
	Placebo	18	Oral	-	-	-
(Venturini et al., 1989) [42]	Gestrinone	11	Oral	2.5 mg	Twice weekly	6 months
(Vercellini et al., 1996) [43]	Gestrinone	27	Oral	2.5 mg	Twice weekly	6 months
	IM LA	28	Intramuscular	3.75 mg	Every 4 weeks	6 months
(Wu et al., 2010) [44]	Gestrinone	498	Oral	2.5 mg	NR	NR
	Mifepristone	498	Oral	2.5 mg	NR	NR
(Xue et al., 2016) [46]	Triptorelin	50	Intramuscular	3.75 mg	Once month	3 months
	Gestrinone	50	Oral	2.5 mg	Twice weekly	6 months
	Mifepristone	50	Oral	25 mg	Twice daily	3 months
(Xue et al., 2018) [45]	Gestrinone	75	Oral	2.5 mg	Twice weekly	24 weeks

NR: not reported. N: number of patients (women).

**Table 4:** Main adverse events associated with the use of gestrinone.

Human Body System	Pharmacological Classification	(%) of Patients
Nervous system	Headache	142/1274 (11.15%)
	Nausea	153/1054 (14.52%)
	Dizziness	73/804 (9.08%)
	Nervousness	29/399 (7.27%)
	Depression	6/150 (4.00%)
Endocrine system	Hirsutism	118/1037 (11.38%)
	Acne and seborrhea	111/260 (42.69%)
	Chloasma	7/302 (2.32%)
Female reproductive system	Amenorrhea	104/251 (41.43%)
	Increased libido	30/249 (12.05%)
	Decreased libido	61/230 (26.52%)
	Hot flushes	85/346 (24.57%)
	Abdominal pain	69/900 (7.67%)
Others	Hoarseness	31/887 (3.49%)
	Cramps	66/355 (18.59%)

**Figure A7:**
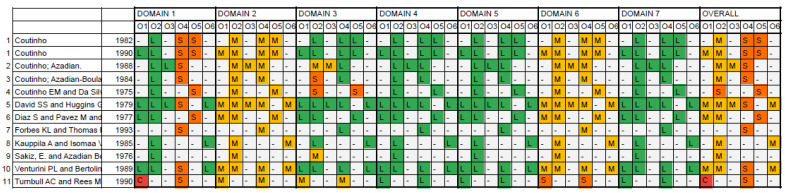
Results of the quality assessment of studies by domain (Robins-I). Legend: risk of bias assessment for each Robins-I domain: confounding (DOMAIN 1); selection bias (DOMAIN 2); bias in measurement classification of interventions (DOMAIN 3); bias due to deviations from intended interventions (DOMAIN 4); bias due to missing data (DOMAIN 5); bias in measurement of outcomes (DOMAIN 6); and bias in selection of the reported result (DOMAIN 7). Outcomes: neurologic system effects (O1); endocrine system effects (O2); reproductive system effects (O3); changes in weight (O4); changes in breast size (O5); and biochemical changes (O6). Risk levels: low (L) (green); moderate (M) (yellow); severe (S) (orange); and critical (C) (red).

## Data Availability

The data supporting the findings of this study are available on Supplementary Materials (https://www.mdpi.com/article/10.3390/pharmaceutics17050638/s1).

## Supplementary Materials

The following supporting information can be downloaded at: https://www.mdpi.com/article/10.3390/pharmaceutics17050638/s1, Table S1: Data extraction table (Excel table), Table S2: Reasons for exclusion.

## Appendix A

**Table A1:** Search strategies.

PubMed	Embase	Web of Science
gestrinone[MH] OR gestrinone[TIAB] OR ‘r2323’[TIAB] OR dimetriose[TIAB] OR nemestran[TIAB] OR ethylnorgestrienone[TIAB]	‘gestrinone‘/exp OR gestrinone:ab,ti OR r2323:ab,ti OR dimetriose:ab,ti OR nemestran:ab,ti OR ethylnorgestrienone:ab,ti	ALL = (gestrinone OR r2323 OR dimetriose OR nemestran OR ethylnorgestrienone)
Total: 308	Total: 745	Total: 180
