# Supplementary material for: Safety Profile of Gestrinone: A Systematic Review
Source: Pharmaceutics. 2025 May 11;17(5):638. doi: 10.3390/pharmaceutics17050638 (PMC12115034; doi:10.3390/pharmaceutics17050638)
Supplement: Supplementary file 1 [file pharmaceutics-17-00638-s001.zip › Table S2.pdf]

Supplementary material
Table S2.

Safety profile of gestrinone: A systematic review

Vitor Luis Fagundes; Nathália Carolina Barreiro Marques; Amanda Franco de Lima; Raul Edison Luna Lazo; Fernanda Stumpf Tonin; Roberto Pontarolo.

Table S2. Excluded studies, after full-text reading, and justifications.

Authors	Article's title	Year of publication	Reason for exclusion
Abrão, M.S. and Podgaec, S. and Carvalho, F.M. and Pinotti, J.A.	Endometriosis in the presacral nerve	1999	wrong drug
Adamo PF and Cantile C and Steinberg H	Evaluation of progesterone and estrogen receptor expression in 15 meningiomas of dogs and cats.	2003	wrong study design
Adashi EY	The morning after: novel hormonal approaches to postcoital interception.	1983	wrong study design
Aguilar, A. and Furio-Bacete, V. and Montalvo, J. and Herraiz, M.	Hyperestrinism and GnRH analogs	1993	wrong outcome
Akinla O and Lähteenmäki P and Jackanicz TM	Intravaginal contraception with the synthetic progestin, R2323.	1976	wrong outcome
Andreeva EN and Iarotskaia EL and Tkachenko ER and Khachatrian AK	[Antihormones and laparoscopy in the combined treatment of disseminated forms of genital endometriosis externa].	1995	foreign language
Angelini, A. and Brusco, G.F. and Arena, S.	Adenomyosis and infertility: Misunderstood pathology?	2005	wrong study design
Anselmo JG	[Hormone regulation of male fertility].	1975	wrong outcome
Arakawa S and Kambegawa A and Okinaga S and Arai K	Luteolytic effect of the antiprogestin and antiglucocorticoid agent RU486 in rats.	1990	wrong study design
Arakawa S and Mitsuma M and Iyo M and Ohkawa R and Kambegawa A	Inhibition of rat ovarian 3 beta-hydroxysteroid dehydrogenase (3 beta-HSD), 17 alpha-hydroxylase and 17,20 lyase by progestins and danazol.	1989	wrong study design

and Okinaga S and Arai K			
Aubeny, E.	Emergency contraception	2000	wrong study design
Azadian Boulanger, G. and Raynaud, J.P. and Gautray, J.P.	Interference of midcycle administration of R2323 with endogenous progesterone	1977	wrong study design
Azadian Boulanger, G. and Secchi, J. and Laraque, F.	Action of a midcycle contraceptive (R 2323) on the human endometrium	1976	wrong study design
Bahgat MR and Atkinson LE	Contraceptive steroid administration by subdermal implants: serum concentrations of R-2323, estrogen and progesterone in rhesus monkeys.	1977	wrong study design
Bai Z	[The use of different dosages, preparations, and combinations of d115-methyl PGF 2alpha for the termination of early pregnancy (132 cases of clinical investigations)].	1986	foreign language
Bartsch G and Wagner I and Rohr HP	The effect of a synthetic progestine on the fine structure of the epididymal head (stereological analysis).	1977	wrong publication type
Battista Candiani, G. and Vercellini, P. and Fedele, L. and Nava, S. and Fontana, P.E.	Medical treatment of mild endometriosis associated with infertility	1991	wrong publication type
Baulieu, E.E.	Antiprogesterone effect and midcycle (periovulatory) contraception	1975	wrong drug
Begum, M.R.	Abnormal uterine bleeding	2021	wrong study design
BeLieu, R.M.	Mastodynia	1994	wrong outcome
Bergquist, A. and Landgren, B.-M. and Diczfalusy, E.	A clinical pharmacokinetic and pharmacodynamic study of three progestogens in the treatment of endometriosis	1988	wrong outcome
Bouchard Ph., and Schaison, G.	Male contraception	1982	wrong publication type
Bouton, M.M. and Martin, P.M. and Raynaud, J.P.	The anti-estrogenic activity of progestins	1979	wrong study design
Bouw, G.M.	The treatment of endometriosis	1990	wrong publication type
Brosens IA and Verleyen A and Cornillie F	The morphologic effect of short-term medical therapy of endometriosis.	1987	wrong study design
Brosens, I.	Strategies for the treatment of endometriosis associated with inofertility and pelvic pain	1988	wrong study design

Brosens, I. and Puttemans, P.	Endometriosis	1994	wrong publication type
Butt, A.M. and Davis, C.	Malignant intracerebral tumours - Long-term remission of recurrences treated with novel therapy	1998	wrong study design
Buttram Jr., V.C.	The rationale for use of medical suppressive therapy prior to endoscopic surgery	1994	wrong study design
Candiani, G.B. and Vercellini, P. and Fedele, L.	Clinical staging and therapeutic choices in endometriosis	1986	wrong study design
Cao Y and Ye Q and Zhuang M and Xie S and Zhong R and Cui J and Zhou J and Zhu Y and Zhang T and Cao L	Ginsenoside Rg3 inhibits angiogenesis in a rat model of endometriosis through the VEGFR-2-mediated PI3K/Akt/mTOR signaling pathway.	2017	wrong study design
Cao Y and Zhuang MF and Yang Y and Xie SW and Cui JG and Cao L and Zhang TT and Zhu Y	Preliminary study of quercetin affecting the hypothalamic-pituitary-gonadal axis on rat endometriosis model.	2014	wrong study design
Carenza, L. and Lopizzo, P. and Terenzi, S. and Montanino, G.	Therapy of non-neoplastic ovarian cysts	1989	wrong study design
Cassidy LM and Moriarty PA and Griffin JF and Kennedy SM	Hormonal treatment of bilateral optic nerve meningioma.	1997	wrong publication type
Cebesoy FB and Kutlar I	Preoperative treatment for hysteroscopic surgery.	2006	wrong publication type
Cerósimo, M.G. and Micheli, F.	Paroxysmal dyskinesia and gonadal sex hormones	1999	wrong publication type
Cervantes Villarreal E and García Zamarripa HR and Herrera Prado E and Barrón Vallejo J	[Gestrinone in pelvic endometriosis. A one-year evaluation].	1995	wrong study desing
Cervantes Villarreal Fr., E. and Garcia Zamarripa, H.R. and Herrera Prado, E.H. and Barron Vallejo, J.	Gestrinone on pelvic endometriosis. Evaluation after one year treatment	1995	Report not retrieved
Chavez, N.F. and Stewart, E.A.	Medical treatment of uterine fibroids	2001	wrong study design
Chen CC and Huang CY and Shiu	Combinatory effects of current regimens and Guizhi Fuling Wan on the development of endometriosis.	2022	wrong study design

LY and Yu YC and Lai JC and Chang CC and Fu CF and Huang SJ			
Chen JW and Tong RX and Yang J and Li QX and Ma HR and Du HL	[Effects of Bushen Wenyang Huayu Recipe on TRPV1 and Sensitization Factor NGF in Experimental Endometriosis].	2015	foreign language
Chen YH and Mao HY and Wu QS and Zhang XH and Shen J and Feng P and Huang CC and Ji XJ	[Mechanism of Shaofu Zhuyu Decoction in treatment of endometriosis-associated dysmenorrhea with syndrome of cold coagulation and blood stasis based on MSK1/2].	2022	foreign language
Chen, N. and Zhu, L. and Lang, J. and Liu, Z. and Sun, D. and Leng, J. and Fan, Q. and Zhang, H. and Cui, Q.	The clinical features and management of perineal endometriosis with anal sphincter involvement: A clinical analysis of 31 cases	2012	wrong study design
Cheng W and Shan J and Ding J and Liu Y and Sun S and Xu L and Yu C	Therapeutic effects of Huayu Jiedu formula on endometriosis via downregulating GATA 6 expression.	2024	wrong study design
Chu, Y.H. and Li, Q. and Zhao, Z.F.	Antiprogestational action of 5α-dihydronorethisterone	1985	foreign language
Ciou HH and Lee TH and Wang HC and Ding YR and Tseng CJ and Wang PH and Tsai MH and Tzeng SL	Repurposing gestrinone for tumor suppressor through P21 reduction regulated by JNK in gynecological cancer.	2022	wrong study design
Cirstoiu, C. and Cirstoiu, M. and Secara, D. and Munteanu, O. and Bodean, O.	Bone mineral density loss associated with low estrogen state induced by gonadotrophin-releasing hormone analogues for endometriosis treatment	2013	wrong publication type
Cornillie FJ and Brosens IA and Vasquez G and Riphagen I	Histologic and ultrastructural changes in human endometriotic implants treated with the antiprogesterone steroid ethylnorgestrienone (gestrinone) during 2 months.	1986	wrong publication type
Cornillie FJ and Vasquez G and Brosens I	The response of human endometriotic implants to the anti-progesterone steroid R 2323: a histologic and ultrastructural study.	1985	wrong study design
Costa De Medeiros, A.G. and De Carvalho Filho, I.R. and Sobral Filho, J.F.	Acneform eruptions due to gestrinona	1996	wrong study design

COUNTINHO, E and SOUZA, JCD and MELO, J	CONTRACEPTIVE EFFECT OF ETHYLNORGESTRIENONE IN MEN AND WOMEN - UNISEX BIRTH-CONTROL PILL	1975	wrong study design
Coutinho E	The vaginal contraceptive pill.	1985	wrong study design
Coutinho E and Gonçalves MT and Azadian-Boulanger G and Silva AR	Endometriosis therapy with gestrinone by oral, vaginal or parenteral administration.	1987	wrong study design
Coutinho EM	Conservative treatment of uterine leiomyoma with the antiestrogen antiprogesterone R-2323.	1981	wrong outcome
Coutinho EM	Therapeutic experience with gestrinone.	1990	wrong publication type
Coutinho EM	GESTRINONE IN THE TREATMENT OF MYOMAS.	1989	wrong outcome
Coutinho, E.M. and Melo, J.F.	Successful inhibition of spermatogenesis in man without loss of libido: a potential new approach to male contraception	1973	wrong outcome
Coutinho, EM	The future of gestrinone	1995	wrong publication type
Creatsas G	Progestogens in reproductive endocrinology.	1991	wrong study design
Crha, I. and Ventruba, P. and Petrenko, M.	Management of leiomyomas	1996	wrong study design
Croxatto HB and Diaz S and Pavez M	Clinical chemistry in women treated with progestogen implants.	1978	wrong outcome
Cunningham D and Gazet JC and Ford HT and Coombes RC	Oral gestrinone: a novel antiprogestin with no antitumor activity in endocrine-sensitive breast cancer.	1987	wrong study design
Dan, W. and Yiling, J. and Chun, L. and Jing, F. and Huimin, W. and Xiaoxin, Y.	Withaferin a downregulates cox-2/nf-κb signaling and modulates mmp-2/9 in experimental endometriosis	2021	wrong study design
Daniel Jr., J.C. and Booher, C.B.	Induction of blastokinin synthesis by R2323 in ovariectomized rabbits	1975	wrong publication type
Davis C	Surgical and non-surgical treatment of symptomatic intracranial meningiomas.	1995	wrong study design
Davis, C.	Meningiomas and sex hormones	1990	wrong publication type
Dawood MY and Khan-Dawood FS and Ramos J	Plasma and peritoneal fluid levels of CA 125 in women with endometriosis.	1988	wrong study design
Deftos LJ	Games of hormones: the para-endocrinology of sport.	2006	wrong publication type

Delettre, J. and Mornon, J.P. and Lepicard, G.	Lack of receptor binding specificity of steroids related to their conformational mobility	1978	wrong study design
Díaz S and Croxatto HB and Pavez M and Quinteros E and Carrillo D and Simonetti L and Croxatto HD and Rosati S	Ectopic pregnancies associated with low dose progestagen-releasing IUDs.	1980	wrong outcome
Doherty, H.A. and Owen, D.	Gestrinone in cyclical breast pain.	1992	wrong publication type
Donadio, N and Nobrega, AC	Leiomyomata plus endometriosis: Simultaneous treatment with gestrinone and buserelin acetate before in vitro fertilization-embryo transfer	1995	wrong study design
Donnez J and Nisolle M and Clerckx F and Casanas F	Evaluation of preoperative use of danazol, gestrinone, lynestrenol, buserelin spray and buserelin implant, in the treatment of endometriosis associated infertility.	1990	Report not retrieved
Donnez J and Nisolle-Pochet M and Casanas-Roux F	Endometriosis-associated infertility: evaluation of preoperative use of danazol, gestrinone, and buserelin.	1990	Report not retrieved
Dorn, SB and Bolt, HM and Degen, GH	The anabolic steroids trenbolone and tetrahydro-gestrinone induce micronuclei in V79 cells	2006	wrong drug
Dowsett M and Forbes KL and Rose GL and Mudge JE and Jeffcoate SL	A comparison of the effects of danazol and gestrinone on testosterone binding to sex hormone binding globulin in vitro and in vivo.	1986	wrong study design
Doyle LL	Hormone-releasing silicone-rubber intrauterine contraceptive devices. Effect of incorporation of various compounds on intrauterine contraceptive devices in rats.	1975	wrong study design
Duan H and Wang S and Hao M and Chen L and Tang J and Wang X and Peng YZ and Zhang SC and Cao LR and Yu JJ	[Research of gestrinone-related abnormal uterine bleeding and the intervention in the treatment: a multi-center, randomized, controlled clinical trial].	2016	foreign language
ElSherif, A. and Valente, S.A.	Management of Mastalgia	2022	wrong publication type
Erickson BE	Detecting doping.	2005	wrong publication type
Fang, M. and Xu, K. and Zhang, W.	Mechanisms of reversible apoptosis in ovarian follicles: Recovery of ovarian reserve after drug use	2008	wrong publication type
Fedele L and Bianchi S and	Histological impact of medical therapy--clinical implications.	1995	wrong study design

Marchini M and Di Nola G			
Fedele L and Marchini M and Baglioni A and Dell'Antonio G and Motta T	Evaluation of histological and ultrastructural aspects of endometrium during treatment with gestrinone in women with amenorrhea or spotting.	1990	wrong study design
Frick J and Bartsch G and Jakse G	Radioimmunoassays of ethinyl-norgestrienone (R-2323) and medroxyprogesterone acetate (MPA) and their clinical applicability.	1977	wrong study design
Frick, J.	Male contraception	1977	wrong publication type
Frishman, G.N. and Jurema, M.W.	Myomas and myomectomy	2005	wrong publication type
Fukuda Y and Tamura S and Hatanaka S	[Effects of gestrinone on serum lipid and lipoprotein levels in women with endometriosis].	1989	foreign language
Furuta, N.	Studies on progesterone receptor in rabbit uterus. Chromatin binding and chromatin template activation	1978	foreign language
Gao X and Wu E and Chen G	Mechanism of emergency contraception with gestrinone: a preliminary investigation.	2007	wrong study design
Garcia, F.L. and Ortega, R.D. and Kably, A.A. and Hernandez, A.V.	Intestinal endometriosis as an acute surgical disease	1997	wrong study design
Giu Y and Huang S and Wu X	[Effects of gestrinone on experimental endometriosis in rabbits].	1995	foreign language
Göretzlehner G and Köhler G	[Antiprogestins].	1988	wrong publication type
Gui, Y.-T. and Wu, C.-J. and Wu, X.-R.	Affinity of gestrinone to estrogen and progestin receptors of uterine cytosol in rabbits	1997	foreign language
Guy, M.M. and Ying, W.Z. and Yan, W.X. and Hui, F.Z. and Hai, W.X. and Ping, L.Y. and Chun, Z.Y. and Aurelie, K.K. and Tao, W.Z.	Effectiveness of treatment for infertility using clinical investigation of laparoscopy cytoreductive surgery combined with gestrinone in adenomyosis	2016	wrong study design
Halbe HW and Nakamura MS and Da Silveira GP and Carvalho WP	Updating the clinical experience in endometriosis--the Brazilian perspective.	1995	wrong outcome
He, K. and Liu, J. and Li, Q. and Li, C.-X. and Liu, L.-H.	Effects of Dan'e Fukang decocted extract on the expression of MMP-9 and TIMP-1 mRNA in endometriosis rats	2012	foreign language
Herremans, D.J.J. and Delaere, K.P.J.	Urinary endometriosis. Three case reports	1995	wrong outcome

Herrera Suastegui, T. and Garza Evia, A.G. and Reyes Cuervo, H. and Alvarado Duran, A.	Agonists of GnRH in gynecology I	1992	wrong study design
Herxheimer A	Gestrinone in cyclical breast pain.	1992	wrong study design
Herxheimer, A. and Doherty, H.A. and Owen, D. and Peters, F.	Gestrinone in cyclical breast pain [13]	1992	wrong publication type
Hess R and Cunill E and Fuchtner C and Alam V and Romero C and Miranda C	[The gestrinone (R-2323) treatment of endometriosis].	1992	Report not retrieved
Hooley RD and Williams DM and Findlay JK	The effect of various steroids on corpus luteum function.	1977	wrong study design
Howard, F.M.	Chronic pelvic pain in women	2001	wrong outcome
Hughes, E. and Brown, J. and Collins, J.J. and Farquhar, C. and Fedorkow, D.M. and Vandekerckhove, P.	Ovulation suppression for endometriosis	2007	wrong study design
Imbesi, G. and Azzerboni, A. and Bonanno, S. and Zoccali, G. and Triolo, O.	Hysteroscopic myomectomy: Long-term results on menstrual patterns and reproductive outcome	2008	wrong drug
Ishchenko IG and Mishchenko AL and Landekhovskiĭ IuD and Ezhova LS	[Changes in the hemostatic system in anomalous uterine hemorrhages and uterine myoma at perimenopause].	1993	foreign language
Jin YC and Liu JX and Wang XF	[Clinical study of the termination of early pregnancy by administration of DL-15 methyl prostaglandin F2a methyl ester in combination with testosterone propionate or mestrinone (R2323)].	1987	foreign language
Jingwei C and Huilan D and Ruixiao T and Hua Y and Huirong M	Effect of Bushenwenyanghuayu decoction on nerve growth factor and bradykinin/bradykinin B1 receptor in a endometriosis dysmenorrhea mouse model.	2015	wrong study design
Johansson, E.D.B. and Luukkainen, T. and Vartiainen, E. and Victor, A.	The effect of progestin R 2323 released from vaginal rings on ovarian function	1975	wrong study design

Kauppila AJ and Telimaa S and Rönnberg L	Steroidal drugs in endometriosis.	1989	wrong publication type
Kelly PA and Asselin J and Turcot-Lemay L and Labrie F and Raynaud JP	Effects of progesterone and R2323 on the development of dimethylbenzanthracene-induced mammary tumors.	1979	wrong study design
Kendle KE and Lee B	Investigation of the influence of progesterone on mouse embryo transport by using antiprogestational steroids.	1980	wrong study design
Kendle, K.E.	Biological evaluation of anti-progestational agents	1979	wrong publication type
Kharbanda SM and Band V and Murugesan K and Farooq A	Modulation of steroid production in goat ovarian cells: effect of progestins and antiprogestins.	1990	wrong drug
Kiesel L and Bertges K and von Holst TR and Runnebaum B	[Treatment of endometriosis].	1989	wrong publication type
Kiriushchenkov AP	[Nemestran (gestrinone) in the treatment of endometriosis].	1994	foreign language
Kitawaki J	[Studies on estradiol dehydrogenase activity in the human uterine endometrium].	1987	foreign language
Kitawaki J and Yamamoto T and Okada H	Induction of estradiol dehydrogenase activity in human uterine endometrium by synthetic steroids.	1988	wrong study design
Kolibianakis, E.M. and Tarlatzis, B.C. and Awwad, J. and Lipari, C.W. and Muasher, S.J.	Is it of value to treat endometriosis prior to IVF?	2006	wrong publication type
Koninckx, P.R.	Drug treatment of endometriosis	1996	wrong study design
La Marca A and Giulini S and Vito G and Orvieto R and Volpe A and Jasonni VM	Gestrinone in the treatment of uterine leiomyomata: effects on uterine blood supply.	2004	wrong study design
Lévesque JF and Templeton E and Trimble L and Berthelette C and Chauret N	Discovery, biosynthesis, and structure elucidation of metabolites of a doping agent and a direct analogue, tetrahydrogestrinone and gestrinone, using human hepatocytes.	2005	wrong publication type

Li Y and Zhu HL and Liang XD and Zhang C and Wang ZH and Cui H	[Outcome analysis of stage III - IV endometriosis after conservative surgery].	2007	foreign language
Li, Y and Wang, Y and Huang, J and Xu, D and Wang, L and Zhao, Y	Apoptosis induced by gestrinone and mifepriston in endometriosis.	2002	wrong study design
Lian F and Liu HP and Wang YX and Zhang JW and Sun ZG and Ma FM and Zhang N and Liu YH and Meng Q	Expressions of VEGF and Ki-67 in eutopic endometrium of patients with endometriosis and effect of Quyu Jiedu Recipe on VEGF expression.	2007	wrong outcome
Lobo VL and Soares JM Jr and de Jesus Simões M and Simões Rdos S and de Lima GR and Baracat EC	Does gestrinone antagonize the effects of estrogen on endometrial implants upon the peritoneum of rats?	2008	wrong study design
Ma JJ and Chen BL and Ma XD and Cao YX	[Effect of gestrinone on growth and apoptosis in isolated ectopic endometrium cells in vitro].	2005	foreign language
Magri B and Viganò P and Rossi G and Somigliana E and Gaffuri B and Vignali M	Comparative effect of the calcium antagonist verapamil and the synthetic steroids gestrinone and danazol on human monocyte phagocytosis in vitro.	1997	wrong study design
Maia, H. and Haddad, C. and Dos Santos Junior, W.S.D. and De Moura Hirsch, M.C. and Casoy, J.	The use of vaginal gestrinone in Pentravan™ for the treatment of endometriosis-related pain	2014	duplicata
Maia, H. and Haddad, C. and Dos Santos, W.S.D. and Casoy, J.	The use of vaginal gestrinone in pentravan™ for the treatment of deep endometriosis-related pain	2015	wrong study design
Marchini M and Fedele L and Bianchi S and Di Nola G and Nava S and Vercellini P	Endometrial patterns during therapy with danazol or gestrinone for endometriosis: structural and ultrastructural study.	1992	wrong study design
Markiewicz L and Gurpide E	Estrogenic and progestagenic activities coexisting in steroidal drugs: quantitative evaluation by in vitro bioassays with human cells.	1994	wrong study design
Markiewicz L and Hochberg RB and Gurpide E	Intrinsic estrogenicity of some progestagenic drugs.	1992	wrong study design
Markiewicz, L. and Gurpide, E.	In vitro bioassays for drugs with dual estrogenic and progestagenic activities	1994	wrong study design

McRobb L and Handelsman DJ and Kazlauskas R and Wilkinson S and McLeod MD and Heather AK	Structure-activity relationships of synthetic progestins in a yeast-based in vitro androgen bioassay.	2008	wrong drug
Mettler L	Medicosurgical treatment of genital endometriosis focusing on gestagens and antigestagens together with surgical pelviscopy.	1991	wrong publication type
Metzker Coutinho, E.	Injectables and implants	1981	wrong study design
Millet, D.	Perspectives in male contraception	1978	wrong study design
Mitreski, A.T. and Cetkovic, N. and Bogavac, M. and Curcic, N. and Ivkovic, B.	Different treatment options of ovarian endometriosis	2010	Report not retrieved
Mizutani T and Sakata M and Miyake A and Tanizawa O and Terada N and Matsumoto K and Terakawa N	No inhibitory effects of gestrinone and medroxyprogesterone acetate on the estrogen production by ovaries of hypophysectomized rats stimulated by gonadotropins.	1992	wrong study design
Mora G and Faundes A and Pastore U	Clinical evaluation of an oral progestin contraceptive, R-2323, 5mg, administered at weekly intervals.	1974	wrong outcome
Mora, G. and Faundes, A. and Johansson, E.D.B.	Lack of clinical contraceptive efficacy of large doses of R 2323 given before implantation or after a missed period	1975	wrong outcome
Mori T and Fujii S and Konishi I	[Pathogenesis and conservative therapy of uterine leiomyoma].	1986	foreign language
Nash, H.A. and Robertson, D.N. and Moo Young, A.J. and Atkinson, L.E.	Steroid release from silastic capsules and rods	1978	wrong study design
Niaraki MA and Moghissi KS and Borin K	The effect of a synthetic progestogen, ethylnorgestrienone, on hypothalamic-pituitary-ovarian function, cervical mucus, vaginal cytology, and endometrial morphology.	1981	wrong outcome
Nieto A and Tacuri C and Serra M and Keller J and Cortés-Prieto J	Evaluation of gestrinone after surgery in treatment of endometriosis.	1997	wrong study design
Nieto Diaz, A. and Tacuri Cevallos, C. and Serra Sevilla, M. and Cortes Prieto, J.	Analysis of tolerance and acceptance to gastrinone in the treatment of endometriosis	1995	wrong study design

Nisolle M and Clerckx F and Casanas-Roux F and Gillerot S and Bourgonjon D and Donnez J	[Treatment of endometriosis. Evaluation of preoperative therapy with danazol, gestrinone and buserelin (nasal spray and implant)].	1990	Report not retrieved
Nisolle-Pochet M and Casanas-Roux F and Donnez J	Histologic study of ovarian endometriosis after hormonal therapy.	1988	wrong study design
O'Brien, J and Berg, S and Pflug, N and Cwiertny, D and Wammer, K	Aqueous photolysis of the steroid gestrinone in the presence of sodium azide	2017	wrong study design
Ohkawa T and Awata S and Arakawa S and Kambegawa A and Okinaga S and Arai K	The inhibitory effect of synthetic steroids on proestrous gonadotropin release in the rat.	1989	wrong study design
Ohno Y and Kitagawa I and Tamura H and Hosoda S and Yamashita S and Honjo H and Okada H	Antiestrogenic effect of gestrinone as an inhibitor of [3H]-estradiol binding to nuclear type II sites.	1991	wrong study design
Ortega FJ and Bañuls MJ and Sanza FJ and Casquel R and Laguna MF and Holgado M and López-Romero D and Barrios CA and Maquieira Á and Puchades R	Biomolecular Interaction Analysis of Gestrinone-anti-Gestrinone Using Arrays of High Aspect Ratio SU-8 Nanopillars.	2012	wrong study design
Peters, F. and Stein, R.C. and Coombes, R.C. and Gazet, J.-C. and Ford, H.T. and Rawson, N.S.B.	Gestrinone in mastalgia: A randomized double bind placebo controlled trial (The Breast 1994; 3: 90-93) [2]	1995	wrong outcome
Petraglia, F. and Luisi, S.	Local drug release systems in endometriosis	2007	wrong study design
Piccione, E. and Noccioli, G.	Actual aspects of hormonal therapy of endometriosis	1987	wrong study design
Polonini, H. and Loures, S. and Alves, M.C. and Ferreira, A.O. and Brandão, M.A.F. and Raposo, N.R.B.	Feasibility study evaluating pentravan® for the intravaginal administration of active pharmaceutical ingredients to reduce pelvic pain related to endometriosis	2018	foreign language

Prentice, A. and Deary, A.J. and Bland, E.	Progestagens and anti-progestagens for pain associated with endometriosis	2001	wrong study design
Presl J	[Gestrinone in the treatment of endometriosis].	1985	foreign language
Presl J	[Additional experience with the treatment of endometriosis using gestrinone].	1988	foreign language
Presl J and Laitl J and Pilka L and Ventruba P	[Gestrinone in the therapy of sterility due to endometriosis].	1992	foreign language
Pruksananonda K and Suwajanakorn S and Boonkasemsanti W and Virutamasen P	Clinical effects of gestrinone for the treatment of pelvic endometriosis in infertile patients.	1999	foreign language
Quereda F and Barroso J and Acién P	Individual and combined effects of triptoreline and gestrinone on experimental endometriosis in rats.	1996	wrong study design
Rabe, T. and Grunwald, K. and Kiesel, L. and Runnebaum, B. and Vecsei, P.	Influence of gestrinon (R 2323) on metabolism with special reference to human adrenal function	1988	foreign language
Rall, H.J.S.	Contraceptive efficacy of 200μg R2323 and 10μg R2858	1981	Report not retrieved
Rasheed Bahgat, M. and Atkinson, L.E. and Brinson, A.O. and Segal, S.J.	Treatment of postpartum rhesus monkeys with progestogen: appearance in milk and effects on lactation	1975	wrong study design
Raynaud, J.P. and Salmon, J. and Azadian Boulanger, G.	Metabolic studies of R 2323, an original contraceptive compound	1973	wrong study design
Reel, J.R. and Humphrey, R.R. and Shih, Y.-H.	Competitive progesterone antagonists: Receptor binding and biologic activity of testosterone and 19-nortestosterone derivatives	1979	wrong study design
Roberts, P.J.	R 2323. Gestrinone (new prop INN)	1980	wrong study design
Rose GL and Dowsett M and Mudge JE and White JO and Jeffcoate SL	The inhibitory effects of danazol, danazol metabolites, gestrinone, and testosterone on the growth of human endometrial cells in vitro.	1988	wrong study design
Rozenbaum, H.	Choosing the right synthetic progestogen	1983	wrong study design
Rozenbaum, H.	The role of synthetic progestatives used alone in contraception	1978	wrong study design

Ruiz-Velasco V and Arceo JR and Armesto A	Comparative efficacy of gestrinone and danazol in infertile women with endometriosis.	1993	Report not retrieved
Saini, S. and Hall, G. and Davis, C.	A large long term radiological study of meningiomas, including the first long term report of anti-progesterone therapy with gestrinone	2012	wrong publication type
Sakiz, E. and Azadian Boulanger, G. and Laraque, F. and Raynaud, J.P.	A new approach to estrogen free contraception based on progesterone receptor blockage by mid cycle administration of ethyl norgestrienone (R 2323)	1974	wrong study design
Sanza FJ and Holgado M and Ortega FJ and Casquel R and López-Romero D and Bañuls MJ and Laguna MF and Barrios CA and Puchades R and Maquieira A	Bio-Photonic Sensing Cells over transparent substrates for anti-gestrinone antibodies biosensing.	2011	wrong study design
Scarth, JP and Clarke, AD and Teale, P and Pearce, CM	Comparative <i>in vitro</i> metabolism of the 'designer' steroid estra-4,9-diene-3,17-dione between the equine, canine and human: Identification of target metabolites for use in sports doping control	2010	wrong study design
Secchi J and Lecaque D	Effects of progestins and antiprogestins on mitochondria in uterine glandular cells in the rat. A quantitative investigation.	1984	wrong study design
SECCHI, J and AZADIANBOULANGER, G and GAUTRAY, JP and RAYNAUD, JP and SAKIZ, E	FINE-STRUCTURE OF EPITHELIAL-CELLS OF HUMAN ENDOMETRIUM AFTER POSTOVULATORY ADMINISTRATION OF ETHYLNORGESTRIENON (R2323)	1975	Report not retrieved
Sharif S and Brennan P and Rawluk D	Non-surgical treatment of meningioma: a case report and review.	1998	wrong publication type
Shen, L. and Li, A. and Li, L. and Wu, Y. and Shen, L. and Jiang, Q. and He, J. and Wu, Z. and Yu, L. and Zhang, X.	Experimental study on the effect of curcumin on endometriosis in rats	2020	foreign language
Sheng, L. and Hao, S.-F. and Zhou, J.-Y. and Yang, Y. and Zhou, X.-Y. and Guo, X.-J. and Li, Z. and Xie, S.-W. and Zhu, Y.	Ginsenoside Rh2 inhibits growth of ectopic endometrium in rat model and its mechanism of action	2013	foreign language
Snyder BW and Beecham GD and Winneker RC	Studies on the mechanism of action of danazol and gestrinone (R2323) in the rat: evidence for a masked estrogen component.	1989	wrong study design

Spellacy WN and Buhi WC and Dumbaugh VA and Birk SA	The effects of a once-a-week steroid contraceptive (R2323) on lipid and carbohydrate metabolism in women during three months of use.	1978	wrong study design
Spellacy WN and Mahan CS and Buhi WC and Dumbaugh VA and Birk SA	Blood glucose, insulin, cholesterol and triglyceride levels in women treated for six months with the weekly oral contraceptive R2323.	1978	wrong study design
Stovall, T.G.	Abdominal and vaginal hysterectomy for uterine myomas: Surgery combined with medical therapy	1992	wrong publication type
Sun XY and Liang Z	Effect of drug therapy on pregnancy rate, sex hormone levels and adverse reactions after laparoscopic surgery in patients with endometriosis and infertility.	2021	wrong publication type
Swerdloff, R.S. and Wang, C. and Bhasin, S.	Developments in the control of testicular function	1992	wrong publication type
Tamaya T and Fujimoto J and Okada H	Treatment of pelvic endometriosis with gestrinone--report on 9 cases.	1985	wrong study design
Tamaya T and Fujimoto J and Watanabe Y and Arahori K and Okada H	Gestrinone (R2323) binding to steroid receptors in human uterine endometrial cytosol.	1986	wrong study design
Tamaya T and Furuta N and Motoyama T and Biku S and Ohono Y and Okada H	Mechanism of antiprogestational action of synthetic steroids.	1978	wrong study design
Tamaya T and Okada H	Receptors--rationales of steroid therapy for pelvic endometriosis.	1987	wrong study design
Tamaya T and Wada K and Imai A and Mori H and Ban H	Rationale for frequency and dose of administration in gestrinone therapy for pelvic endometriosis in the experimental model of rabbit uterus.	1991	wrong study design
Tamaya, T. and Fujimoto, J. and Fujimoto, Y. and Kawabata, I. and Okada, H.	Treatment of pelvic endometriosis with gestrinone - comparative studies on effectiveness in different doses	1987	foreign language
Tang Q and Shang F and Wang X and Yang Y and Chen G and Chen Y and Zhang J and Xu X	Combination use of ferulic acid, ligustrazine and tetrahydropalmatine inhibits the growth of ectopic endometrial tissue: a multi-target therapy for endometriosis rats.	2014	wrong study design
Taylor, P.J. and Kredentser, J.V.	Nonsurgical management of minimal and moderate endometriosis to enhance fertility	1992	wrong study design
Terakawa N	[Studies on endocrine therapy of endometriosis].	1989	foreign language

Thomas EJ	The relevance of asymptomatic endometriosis.	1989	wrong publication type
Thomas EJ and Cooke ID	Successful treatment of asymptomatic endometriosis: does it benefit infertile women?	1987	wrong study design
Thomas, E.J.	Combining medical and surgical treatment for endometriosis: The best of both worlds?	1992	wrong publication type
Tomonari, R. and Sakai, M. and Iino, Y.	Studies of clinical and endocrinological effects of gestrinone on endometriosis	1987	foreign language
Tostes, FM and Carneiro, F and Amorim, G	Effects of the exposure of different cell lines to the steroidal hormone gestrinone	2021	wrong study design
Tredway DR and Mishell DR Jr	Effect of a once weekly oral contraceptive upon gonadotrophin and gonadal steroid levels.	1974	wrong study design
Utsunomiya T and Sumioki H and Korenaga S and Matsuoka K and Korenga M and Kadota T	[Laparoscopic diagnosis and evaluation of danazol or gestrinone therapy for endometriosis in sterility].	1988	foreign language
Verma U and Laumas KR	Screening of anti-progestins using in vitro human uterine progesterone receptor assay system.	1981	wrong study design
Victor, A. and Johansson, E.D.B.	The effect of weekly administration of an oral progestogen R 2323 on the ovarian function	1975	wrong outcome
Viganò P and Magri B and Di Blasio AM and Busacca M and Vignali M	Gestrinone inhibits macrophage function and mitogen-stimulated lymphocyte proliferation in vitro.	1994	wrong study design
VIINIKKA, L and JOHANSSO.ED and JANNE, O	PLASMA CONCENTRATIONS OF A PROGESTIN R2323 RELEASED FROM VAGINAL RINGS	1974	wrong publication type
Viinikka, L. and Victor, A. and Janne, O. and Raynaud, J.P.	The plasma concentration of a synthetic progestin, R 2323, released from polysilastic vaginal rings	1975	wrong publication type
Wang D and Jiang Y and Yang X and Wei Q and Wang H	6-Shogaol reduces progression of experimental endometriosis in vivo and in vitro via regulation of VGEF and inhibition of COX-2 and PGE2-mediated inflammatory responses.	2018	wrong study design
Wang Q and Zhao H and Xiang Q and Ju H and Han SM and Wang LY and Xu B	Effect of Yikun Neiyi Wan on the expression of aromatase P450, COX-2, and ER related receptor in endometrial cells in vitro from patients with endometriosis.	2009	wrong study design
Wang, P.-H. and Liu, W.-M. and Fuh, J.-L. and Cheng, M.-H. and Chao, H.-T.	Comparison of surgery alone and combined surgical-medical treatment in the management of symptomatic uterine adenomyoma	2009	wrong drug

Wang, R.-Q. and Chen, L. and Cheng, W. and Zhang, J. and Liu, L.-X. and Yao, R.-P. and Ji, X. and Yu, C.-Q.	Neiyi recipe improving reproductive ability of mice with endometriosis and its mechanism	2019	foreign language
Wang, S. and Duan, H.	Research of gestrinone-related abnormal uterine bleeding and the intervention in the treatment: A multi-center, randomized, controlled clinical trial	2016	wrong publication type
Wang, S. and Lang, J.-H. and Leng, J.-H. and Zhu, L. and Fan, Q.-B. and Li, X.-C.	Serum CA125 changes in one case with malignant transformation from benign endometriosis to Müllerian adenosarcoma	2013	wrong publication type
Ward BG and McGuckin MA and Ramm L and Forbes KL	Expression of tumour markers CA125, CASA and OSA in minimal/mild endometriosis.	1991	wrong study design
Worthington M and Irvine LM and Crook D and Lees B and Shaw RW and Stevenson JC	A randomized comparative study of the metabolic effects of two regimens of gestrinone in the treatment of endometriosis.	1993	wrong study design
Wu X and Xu Y	Gestrinone combined with ultrasound-guided aspiration and ethanol injection for treatment of chocolate cyst of ovary.	2015	wrong study design
Xiao, B.	Abortion and emergency contraception: Chinese experience	1997	wrong publication type
Yan, Y. and Li, L. and Guo, J. and Zheng, Y. and Liu, Q.	Malignant transformation of an endometriotic lesion derived from an abdominal wall scar	2011	wrong study design
Yang TS and Tsan SH and Chen CR and Chang SP and Ng HT	The efficacy and safety of a 19 nor-steroid in the treatment of endometriosis.	1996	Report not retrieved
Yang, Y. and Ma, Y.	Efficacy of Goserelin Acetate Sustained-release Depot Combined with Gestrinone in Patient with Endometriosis and its Effect on Oxidative Stress and Inflammatory Response	2022	wrong study design
Yang, Y. and Zhao, R. and Hao, Z. and Li, L. and Xu, C. and Cui, Y.	Effects of danchi decoction on P450arom, survivin of eutopic endometrium of patients with endometriosis after conservative surgery	2015	wrong study design
Yi, X. and Wang, J. and Chang, K. and Xu, H. and Hua, K.	Successful twin pregnancy in a patient with hemi-uterus corrected by laparoscopic Strassman's metroplasty	2017	wrong publication type
Yokoyama, Y. and Niwa, K. and Tamaya, T.	Norethindrone scatters silver-stained nucleolar organizer regions of ishikawa cells	1991	wrong study design
Yoshida K and Otsuka H and	[The effect of a synthetic progestin (R 2323) on gonadal and endometrial cells in vitro and in vivo].	1985	foreign language

Okamura Y and Kadota T			
Yoshimura Y and Maruyama K and Oda T and Shiraki M and Nakamura Y and Kawakami S	Progesterone protects oocytes from premature degeneration within the follicle.	1990	wrong study design
Zhang C and Gao L and Yi Y and Han H and Cheng H and Ye X and Ma R and Sun K and Cui H and Chang X	Adenosine Triphosphate Regresses Endometrial Explants in a Rat Model of Endometriosis.	2016	wrong study design
Zhang C and Zhang X and Li L and Zhou Y	[Clinical effect evaluation of acupuncture combined with medication for prevention of endometriosis recurrence after surgery].	2016	foreign language
Zhang XY and Zhang CY	[Efficacy observation on the combination of acupuncture and Chinese medication in prevention of the recurrence of endometriosis after laparoscopic surgery].	2014	foreign language
Zhang YX	Effect of mifepristone in the different treatments of endometriosis.	2016	wrong outcome
Zhang, JL and Ma, Y and Lian, LL and Wei, M	Clinical Effect of GnRH-a and Gestrinone after Severe Endometriosis Operation: Randomized Clinical Trial	2023	wrong outcome
Zhao RH and Hao ZP and Zhang Y and Lian FM and Sun WW and Liu Y and Wang R and Long L and Cheng L and Ding YF and Song DR and Meng QW and Wang AM	Controlling the recurrence of pelvic endometriosis after a conservative operation: comparison between Chinese herbal medicine and western medicine.	2013	wrong outcome
Zhao RH and Liu Y and Tan Y and Hao ZP and Meng QW and Wang R and Long D and Ding YF and Song DR and Xu C and Ren ZZ and Yang YH and Wang AM	Chinese medicine improves postoperative quality of life in endometriosis patients: a randomized controlled trial.	2013	wrong outcome
Zheng P and Zhang YF and Wang JD	[Effect of gestrinone on the lipid metabolic parameters and bone mineral density in patients with endometriosis].	2005	foreign language
Zhou XH and Fu WJ and Wang BD and Fu QX	[Effect of Tripterygium wilfordii glycosides combined with gestrinone on endometriosis and serum cytokine expression].	2016	foreign language
Zhu Y and Qiu XY and Wang L and Wu JH and Liu GM	Effects of gestrinone on uterine leiomyoma and expression of c-Src in a guinea pig model.	2007	wrong study design

and He GL and Jiang XR and Sun ZY and Cao L			
Zhu Y and Zhang T and Xie S and Tu R and Cao Y and Guo X and Zhou J and Zhou X and Cao L	Gestrinone inhibits growth of human uterine leiomyoma may relate to activity regulation of ERα, Src and P38 MAPK.	2012	wrong study design
Zhu, Y and Qiu, XY and Wang, L and Wu, JH and He, GL and Liu, GM and Jiang, XR and Sun, ZY and Cao, L	Effect of gestrinone on gene expression in human uterine leiomyoma	2006	foreign language
Zhuang M and Cao Y and Shi Y and Yu L and Niu Y and Zhang T and Sun Z	Caulis Sargentodoxae Prescription Plays a Therapeutic Role with Decreased Inflammatory Cytokines in Peritoneal Fluid in the Rat Endometriosis Model.	2020	wrong study design
Zhuang, M.-F. and Cao, Y. and Shi, Y. and Yu, L. and Niu, Y.-N. and Zhang, T.-T. and Sun, Z.-G.	Caulis sargentodoxae prescription inhibits angiogenesis-related cytokines in a rat endometriosis model	2017	wrong study design
Zikopoulos, K. and Kolibianakis, E.M. and Devroey, P.	Ovarian stimulation for in vitro fertilization in patients with endometriosis	2004	wrong study design
	Gestrinone (Dimetriose)--another option in endometriosis.	1991	wrong publication type
	Treatment of pelvic pain associated with endometriosis.	2008	wrong study design
	MRCOG part II model essay answer: Evaluate the non-surgical treatments available for symptomatic endometriosis	2004	wrong study design
	Hot on the heels of new drugs in sport: Undetectable or just not yet detected?	2003	wrong study design
	Drug theray of endometriosis	1993	wrong study design
	Gonadotropin releasing hormone analogues for endometriosis	1993	wrong study design
	Androgen in the treatment of mastodynia	1992	wrong study design
	Steroid oximes as contraceptives with antiimplantation and interceptive action	1981	wrong study design
